# CoolTip: Low-Temperature Solid-Phase Extraction Microcolumn for Capturing Hydrophilic Peptides and Phosphopeptides

**DOI:** 10.1016/j.mcpro.2021.100170

**Published:** 2021-11-03

**Authors:** Kosuke Ogata, Yasushi Ishihama

**Affiliations:** 1 Department of Molecular & Cellular BioAnalysis, Graduate School of Pharmaceutical Sciences, Kyoto University, Kyoto, Japan; 2 Laboratory of Clinical and Analytical Chemistry, National Institute of Biomedical Innovation, Health and Nutrition, Ibaraki, Osaka, Japan

**Keywords:** ACN, acetonitrile, PGC, porous graphitic carbon, RP, reversed-phase, SDB, poly(styrene-divinylbenzene) copolymer, StageTip, stop-and-go extraction tip, TiO_2_, titanium dioxide

## Abstract

Reversed-phase solid-phase extraction (SPE) techniques are commonly used for desalting samples before LC/MS/MS in shotgun proteomics. However, hydrophilic peptides are often lost during the desalting step under the standard SPE conditions. Here, we describe a simple protocol in which a stop-and-go extraction tip packed with a poly(styrene-divinylbenzene) copolymer disc is used at 4 °C during sample loading without any organic solvent. Using this method, which we designate as the CoolTip protocol, we identified 2.9-fold more tryptic peptides and 6.1-fold more tryptic phosphopeptides from HeLa lysates than the standard SPE protocol for hydrophilic peptides, with a mobile phase of less than 8% acetonitrile in LC/MS/MS. There was no decrease in the recovery of hydrophobic peptides. CoolTip also provided better quantitative reproducibility in LC/MS/MS analysis. We anticipate that this protocol will provide improved performance in many kinds of shotgun proteomics experiments.

## Highlights

• CoolTip, a StageTip with a poly(styrene-divinylbenzene) disc operated at 4 °C.
• Identification of more 6.1-fold hydrophilic phosphopeptides from HeLa lysates.
• No decrease in the recovery of hydrophobic peptides using the CoolTip protocol.
• Better reproducibility in quantitative LC/MS/MS analysis.

## Graphical Abstract

**Figure:**
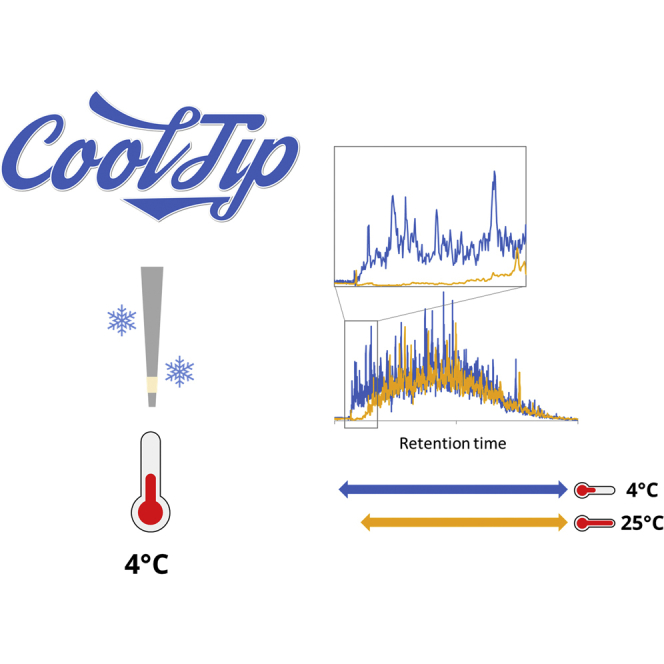

## In Brief

A desalting protocol to increase the recovery of hydrophilic peptides and phosphopeptides was developed by using SDB-StageTip at 4 °C. Using this protocol, 2.9-fold more tryptic peptides and 6.1-fold more tryptic phosphopeptides from HeLa lysates were identified for hydrophilic peptides.

Proteomics methodologies for the comprehensive identification and quantification of expressed proteins and their post-translational modifications have become indispensable for biological research (1). In particular, shotgun proteomics using LC/MS/MS is a reliable and highly sensitive tool for identifying proteins in biological samples (2). In a typical shotgun proteomics workflow, peptides must be concentrated before LC/MS/MS analysis to remove interfering small molecules, not only to increase the efficiency of target peptide ionization but also to extend the lifetime of the analytical system (3). Specifically, peptides are loaded onto reversed-phase (RP) materials, washed with appropriate solutions, and eluted with organic solvents for subsequent LC/MS/MS analysis. On-line trap columns are effective for automating sample preparation and reducing injection time when the sample volume is large (4). However, because the size and properties of the trap column are restricted by the nature of the analytical column used, the size of the trap column should be carefully selected to provide sufficient loading capacity while maintaining separation efficiency (5). Inappropriate selection of trap columns for samples often leads to the loss of hydrophilic peptides (6, 7). On the other hand, it is easier to achieve optimal conditions with off-line solid-phase extraction columns. Currently, solid-phase extraction microcolumns using pipette tips such as stop-and-go extraction tips (StageTips), which consist of small discs of RP beads embedded in a Teflon mesh, are widely used for capturing peptides because of their large capacity, high recovery, and low elution volume required (3, 8). With these tip-based microcolumns, samples can be processed in parallel, thus reducing the total analysis time. However, hydrophilic peptides such as very short peptides and phosphorylated peptides are not well retained in the solid phase and are easily lost during the purification process (9, 10), resulting in insufficient proteome coverage.

Traditionally, carboxylic acids with fluorocarbon chains have been used as ion-pair reagents to improve the retention of hydrophilic peptides on RP columns (11). However, these strong ion-pair reagents significantly reduce the efficiency of ESI, leading to a reduction in the sensitivity of MS analysis (5). Even if ion-pair reagents with long fluorocarbon chains are used only during sample preparation, they can still contaminate subsequent LC/MS analyses (12). In addition, the use of hydrophobic ion-pair reagents is less effective to improve the retention of phosphopeptides, as compared with nonphosphopeptides (13, 14, 15). Chemical modification of peptides with nonpolar groups can enhance the hydrophobicity, improve the retention, and increase the recovery of hydrophilic peptides during sample preparation (10, 16, 17). However, these approaches generally require more complex workflows to label the peptides and are often difficult to apply to limited amounts of samples. So far, porous graphitic carbon (PGC) chromatography seems to be the most promising alternative to RPLC for the analysis of short and hydrophilic peptides (18, 19). However, owing to the low recovery of hydrophobic peptides in PGC chromatography, the implementation of PGC columns always requires the combined use of RP columns, both in on-line and off-line modes (9, 20, 21).

The relationship between solute retention and column temperature in LC is described by Equations 1 and 2 (22).

(1) ΔG°=−RTln(k/Φ) 

(2) lnk=−ΔH°/RT+ΔS°/R+lnΦ

where ΔG° is the change in Gibbs free energy, *k* is the retention factor, Φ is the phase ratio, ΔH° is the change in enthalpy, and ΔS° is the change in entropy for phase transfer from the mobile to the stationary phase. This formula is known as the van't Hoff equation and implies a linear dependence of ln *k* on 1/T, if ΔS°, ΔH°, and Φ is assumed to be independent of the temperature (23). In RPLC/MS analysis, an increase of the column temperature generally leads to shorter elution times of analytes and sharper peak shapes (24). In contrast, decreasing the column temperature can trap hydrophilic solutes more effectively (25, 26), and this leads to better peak shapes based on thermal peak focusing of peptides and proteins (27). It was reported that low-temperature sample loading in RPLC/MS led to higher bovine serum albumin sequence coverage, as well as an increase in the number of identified peptides in proteomic samples (28, 29).

In this study, based on the hypothesis that chilling the RP microcolumn would increase its ability to trap hydrophilic (phospho)peptides during the desalting step, we aimed to develop a simple method to increase the capture of hydrophilic peptides for global and phosphoproteome analysis by using a cooled RP-StageTip, without sacrificing the recovery of hydrophobic peptides.

## Experimental Procedures

### Materials

UltraPure Tris Buffer was purchased from Thermo Fisher Scientific. Sequencing-grade modified trypsin was purchased from Promega. Water was purified by a Millipore Milli-Q system. PGC tips, Empore C8 and poly(styrene-divinylbenzene) copolymer (SDB) extraction disks, and InertSep RP-C18 and InertSep PLS-2 were purchased from GL Sciences. All other chemicals and reagents were purchased from Fujifilm Wako unless otherwise specified.

### Cell Culture

HeLa S3 cells were cultured to 80% confluency in Dulbecco's modified Eagle's medium containing 10% fetal bovine serum in 10-cm diameter dishes. Cells were washed twice with ice-cold PBS, collected using a cell scraper, and pelleted by centrifugation.

### Protein Digestion and Phosphopeptide Enrichment

HeLa cell lysates were digested by means of phase-transfer surfactant–aided trypsin digestion as described previously (30). Briefly, the cell pellets were suspended in 1 ml of the buffer (12 mM sodium deoxycholate, 12 mM sodium lauroyl sarcosinate in 100 mM Tris-HCl, pH 9.0), containing protein phosphatase inhibitor cocktail 1 and 2 (Sigma) and protease inhibitors (Sigma). The cells were incubated on a heating block at 95 °C for 5 min and then sonicated for 20 min. The extracted proteins were quantified with a BCA protein assay kit, reduced with 10 mM DTT for 30 min, and alkylated with 50 mM iodoacetamide for 30 min in the dark. The samples were diluted 5-fold with 50 mM ammonium bicarbonate and then digested with Lys-C for 3 h at room temperature and with trypsin overnight at 37 °C. For global proteome analysis, 1 ml of ethyl acetate was added to 1 ml of the digested solution, and the mixture was acidified with 0.5% TFA (final concentration). The samples were vortexed for 2 min and centrifuged at 15,800*g* for 2 min to completely separate the aqueous and organic phases. The aqueous phase was collected, dried, resuspended to 0.1% TFA/0, 2, or 4% acetonitrile (ACN) solution, and desalted using SDB-StageTips.

For phosphoproteome analysis, phosphopeptide enrichment was performed as described previously, with some modifications (31, 32). In short, 4 ml of ACN, 706 μl of lactic acid, and 28 μl of TFA were added to 1 ml of the digested solution to give final concentrations of 69.8, 12.8, and 0.5%, respectively. C8-StageTips packed with titanium dioxide (TiO_2_) beads (0.5 mg beads/10 μl pipet tip) were equilibrated with 20 μl of 0.1% TFA/80% ACN, containing lactic acid as a selectivity enhancer at a concentration of 300 mg/ml (wash solution). The digested samples (100 μg peptide) were loaded onto the TiO_2_/C8-StageTip. The StageTip was washed with 20 μl of the wash solution and 50 μl of 0.1% TFA/80% ACN, and phosphopeptides were eluted with 50 μl 15% NH_4_OH/40% ACN. The eluted fractions were combined, dried, resuspended to 100 μl of 0.1% TFA/0, 2, or 4% ACN solution, and desalted using SDB-StageTips.

### Low-Temperature StageTip Desalting

SDB-StageTips were manufactured as described previously (8). Briefly, three pieces of the Empore SDB disc were stamped out with a 16G blunt-ended syringe needle (Hamilton) and packed into a 200 μl tip. All procedures including conditioning, sample loading, washing, and elution were performed in the Eppendorf 5415R refrigerated centrifuge. The solvents used for desalting are summarized in Table 1. The cooling of StageTips was accomplished by setting the centrifuge temperature at 4 °C for 5 min. All solvents including the sample solution were prepared at 25 °C and placed on the SDB disc of the desalting tip without precooling. After standing for 1 min, centrifugation was started, and 50 μl of the solution was passed through the tip at 1500*g* for 3 min. The entire protocol is described in supplemental data. After desalting, peptides were dried in a vacuum centrifuge and the residue was dissolved in 4% ACN/0.5% TFA.

**Table 1:** Summary of solvents used for peptide desalting

Step number	Content	4% ACN	2% ACN	0% ACN
Step 1	Wash	80% ACN and 0.1% TFA	80% ACN and 0.1% TFA	80% ACN and 0.1% TFA
Step 2	Equilibration	4% ACN and 0.1% TFA	2% ACN and 0.1% TFA	0.1% TFA
Step 3	Sample load	4% ACN and 0.1% TFA	2% ACN and 0.1% TFA	0.1% TFA
Step 4	Wash	4% ACN and 0.1% TFA	2% ACN and 0.1% TFA	0.1% TFA
Step 5	Elute	80% ACN and 0.1% TFA	80% ACN and 0.1% TFA	80% ACN and 0.1% TFA

### LC/MS/MS Analysis

NanoLC/MS/MS analyses were performed on a Q-Exactive (Thermo Fisher Scientific), which was connected to an UltiMate 3000 pump (Thermo Fisher Scientific) and an HTC-PAL autosampler (CTC Analytics). Peptides were separated on pulled in house needle columns (150-mm length, 100 μm inner diameter, 6-μm needle opening) packed with ReproSil-Pur 120 C18-AQ 3-μm RP material (Dr Maisch). The samples were applied by 5-μl full loop injection, and the flow rate was 500 nl/min. Separation was achieved by applying a three-step linear gradient of 4 to 8% ACN in 5 min, 8 to 32% ACN in 60 min, 32 to 80% ACN in 5 min, and 80% ACN for 5 min with 0.5% acetic acid. Spray voltage was set to 2.4 kV, the ion transfer tube was heated to 250 °C, and S-lens RF level was set at 50. Raw MS1 spectra were collected at a resolution of 70,000. Full-scan automatic gain control target was 3 × 10^6^ with a maximum injection time of 100 ms. The full-scan mass range was set to 350 to 1500. The automatic gain control target value for fragment scans was set at 1 × 10^5^, with a maximum injection time of 100 ms. The orbitrap was operated at 17,500 resolution, and precursors were fragmented by higher-energy collisional dissociation at a normalized collision energy of 27%. The quadrupole isolation width was set to 2 Da, and the top 10 data-dependent MS2 scans were collected between full MS scans. All data were acquired in the profile mode using positive polarity.

### Database Searching and Data Processing

For peptide identification, the peak list in Mascot generic format was generated by MaxQuant version 1.6.17.0 (33). Peptides and proteins were identified by means of automated database searching using Mascot version 2.7.0 (Matrix Science) against the human database from UniProtKB/Swiss-Prot release 2019/10 (20,380 sequences) with a precursor mass tolerance of 5 ppm, a fragment ion mass tolerance of 20 ppm, and strict trypsin/P specificity allowing for up to 2 missed cleavages. Carbamidomethyl (C) was set as a fixed modification. Methionine oxidation was set as a variable modification. For phosphoproteome analysis, methionine oxidation and phosphorylation on serine, threonine, and tyrosine were allowed as variable modifications. Peptides were accepted if the Mascot score was over the 95% confidence limit (*p* < 0.05) based on the identity score of each peptide. False discovery rates of less than 1% estimated by searching against a reversed decoy database were applied for peptide identification.

## Results and Discussion

### Decreasing the StageTip Temperature Extends the Proteome Coverage

**Fig. 1:**
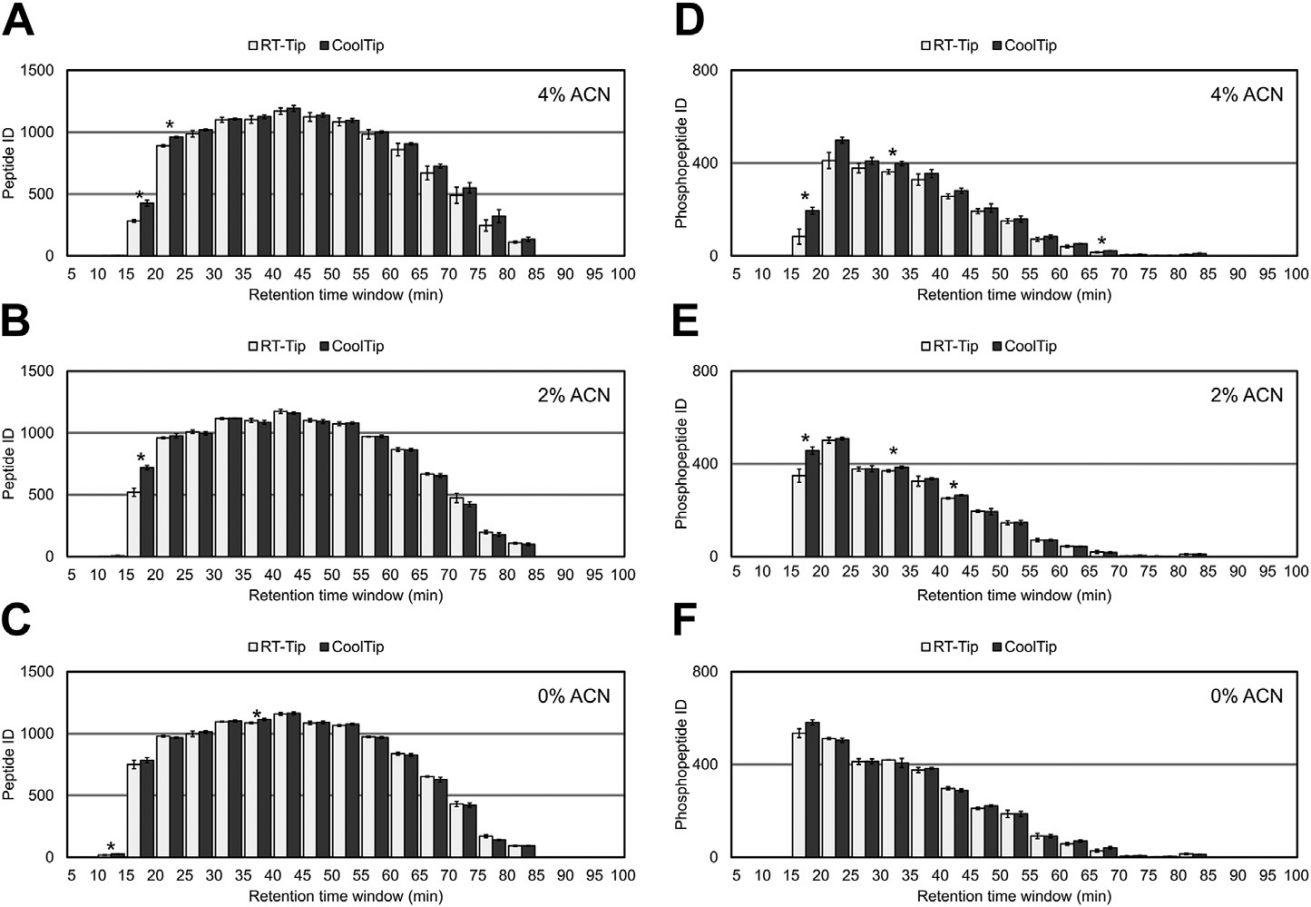
**Comparison of peptide identification numbers binned by retention time.** The *bars* indicate peptide identification numbers in each bin. The error bars indicate the SDs of triplicate analyses. ∗*p* < 0.05. *A–C*, results of global proteomics with (*A*) 4% ACN, (*B*) 2% ACN, and (*C*) 0% ACN solvent for loading and washing at the desalting step. *D–F*, results of phosphoproteomics with (*D*) 4% ACN, (*E*) 2% ACN, and (*F*) 0% ACN solvent for loading and washing at the desalting step. ACN, acetonitrile.

We first compared SDB-StageTip with PGC-StageTip as a desalting microcolumn for the analysis of 20 μg tryptic peptides from HeLa cell lysates and found that SDB-StageTip failed to capture hydrophilic peptides with earlier retention times in LC/MS/MS (<20 min, corresponding to ACN concentrations below 8%), whereas these peptides were successfully captured by PGC-StageTip (Fig. S1*A*). However, the recovery of hydrophobic peptides was lower with PGC-StageTip (Fig. S1*A*), indicating that neither SDB-StageTip nor PGC-StageTip is suitable to capture tryptic peptides having a wide range of hydrophilicity/hydrophobicity. Next, we increased the volume of the stationary phase by adding chromatographic sorbents on top of the SDB-StageTip. We used SDB beads (InertSep PLS-2) or more hydrophobic C18-modified SDB beads (InertSep RP-C18) and examined the recovery of hydrophilic peptides. We also used heptafluorobutyric acid instead of TFA as an ion-pair reagent to increase the hydrophobicity of the sample peptides (34). As shown in Figure S1*B*, neither the more hydrophobic sorbent (SDB-C18) nor the ion-pair reagent (heptafluorobutyric acid) improved the recovery of the hydrophilic peptides. A modest improvement in the recovery of hydrophilic peptides was observed in the case of SDB-StageTip packed with additional SDB beads. However, PGC-StageTip still gave the best recovery of hydrophilic peptides. To improve the recovery further, we next examined the effect of changing the temperature of the SDB-StageTip during sample loading. Because we always use a thermostated centrifuge for desalting steps with StageTip, we set the temperature of the centrifuge to 4 °C and cooled the StageTip in the centrifuge for 5 min before starting the desalting step. We then compared this low-temperature StageTip (CoolTip) protocol with the standard SDB-StageTip protocol (RT-Tip) for the analysis of 5-μg tryptic peptides from HeLa lysates. Three solutions with different contents of ACN (0, 2, or 4% ACN with 0.1% TFA) for the sample loading and washing steps were evaluated because the ACN concentration strongly affects the retention of peptides on RP materials. After desalting, a 500-ng aliquot of peptides was injected onto the nanoLC/MS/MS system. Figure 1, *A*–*C* shows the identification number of peptides binned by retention time for the RT-Tip and CoolTip protocols. The number of peptide identifications was higher in the CoolTip protocol, especially for hydrophilic peptides eluting at less than 20 min (corresponding to ACN concentrations below 8%), with all solvents examined. Thus, recovery of hydrophilic peptides was improved by decreasing the temperature of the StageTip. The temperature effect was greater at higher ACN concentrations. Although 2 to 4% ACN solvents for sample loading and washing steps have been widely used for desalting peptides, the results indicated that hydrophilic peptides were lost during desalting under these conditions. However, because these hydrophilic peptides correspond to only a small proportion of the total number of identified peptides (less than 10%), the total identification numbers were not significantly different across all evaluated conditions.

In addition, we evaluated a series of StageTip temperatures (25 °C, 16 °C, 8 °C, and 4 °C) and found that the recovery of hydrophilic peptides increased as the tip temperature was lower (Fig. S2). Furthermore, we evaluated the precooling effect of the loading, wash, and elution solutions on the recovery of hydrophilic peptides in the CoolTip desalting at 4 °C and found no difference between the solutions with and without precooling (Fig. S2). Based on these results, we concluded that the maximum recovery can be obtained at 4 °C and the solutions do not need to be precooled.

**Fig. 2:**
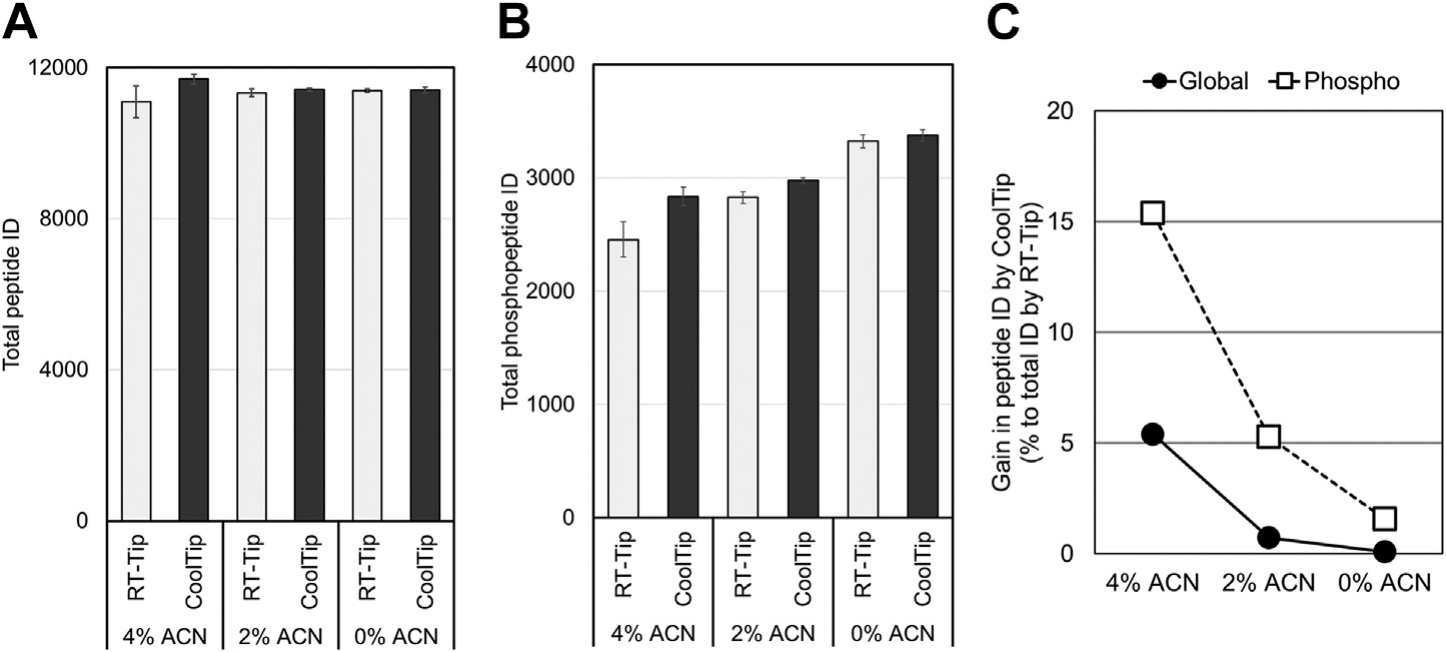
**Summary of total peptide identifications in each condition.***A*, total peptide identifications in global proteomics experiments. *B*, total peptide identifications in phosphoproteomics experiments. The bar plots show the peptide identification numbers. The error bars show the SDs from triplicate analyses. *C*, the gain in peptide identifications achieved by the CoolTip strategy. The average peptide identification numbers in CoolTip were compared with the average peptide identification numbers in RT-Tip under each solvent condition. ACN, acetonitrile.

Generally, phosphopeptides are more hydrophilic than nonphosphopeptides because the additional phosphate group increases the hydrophilicity. Therefore, we hypothesized that the enhancement of hydrophilic peptide recovery by CoolTip would be particularly advantageous for phosphoproteome analysis. Usually, the phosphoproteomics sample preparation workflow requires two desalting steps, one before phosphopeptide enrichment and one after phosphopeptide enrichment. Here, for simplicity, only a single desalting procedure was incorporated into the phosphoproteomics workflow by utilizing a lysis buffer compatible with phosphopeptide enrichment (32, 35). In short, a 30-μg aliquot of digested peptides was directly subjected to TiO_2_ chromatography, and the eluent containing approximately 150 ng of phosphopeptides was desalted using a StageTip and analyzed by LC/MS/MS. Figure 1, *D*–*F* shows the identification number of phosphopeptides binned by retention time. Phosphopeptide elution windows are shifted earlier than those of nonphosphopeptides, resulting in higher percentages of identified hydrophilic phosphopeptides. Similar to the case of nonphosphopeptides, more phosphopeptides were identified especially at earlier retention times (<20 min) in the CoolTip protocol. The temperature effect was also more prominent at higher ACN concentrations for sample loading/washing steps, as was the case for nonphosphopeptides. The total numbers of the identified phosphopeptides were more sensitive to the temperature, as well as the ACN concentration of the loading buffer, than those of nonphosphopeptides, indicating that the CoolTip protocol is more effective for phosphopeptides. In summary, in solvents with all the ACN concentrations examined, the CoolTip protocol provided more identifications than the RT-Tip protocol for both global proteome and phosphoproteome samples (Fig. 2, *A*–*C*). Finally, the optimized CoolTip protocol was compared with the PGC-tip protocol (18, 21). Again, CoolTip provided better identification numbers for both hydrophilic and hydrophobic (phospho)peptides (Fig. S3). All identified peptides are summarized in supplemental data, located in the jPOST repository with PXD028871.

### Low-Temperature StageTip Enhanced the Quantitative Performance of the Analysis

**Fig. 3:**
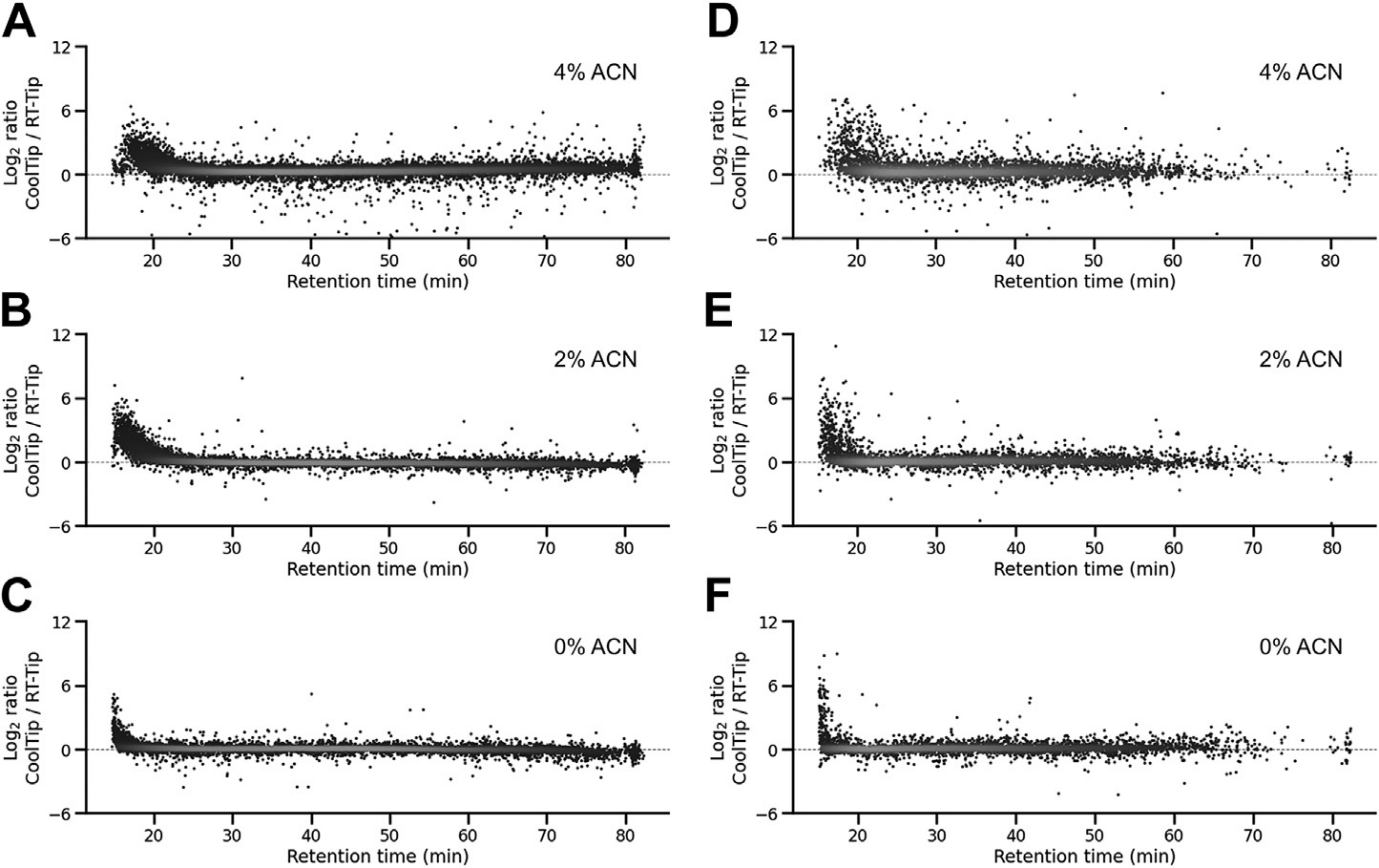
**Relative quantitation results for CoolTip and RT-Tip as a function of peptide retention time.** MS1 label-free quantitation was performed for identified peptides in each solvent condition. Each *dot* represents a peptide quantitative ratio. *A–C*, results of global proteomics with (*A*) 4% ACN, (*B*) 2% ACN, and (*C*) 0% ACN solvent for loading and washing at the desalting step. *D–F*, results of phosphoproteomics with (*D*) 4% ACN, (*E*) 2% ACN, and (*F*) 0% ACN solvent for loading and washing at the desalting step. ACN, acetonitrile.

**Fig. 4:**
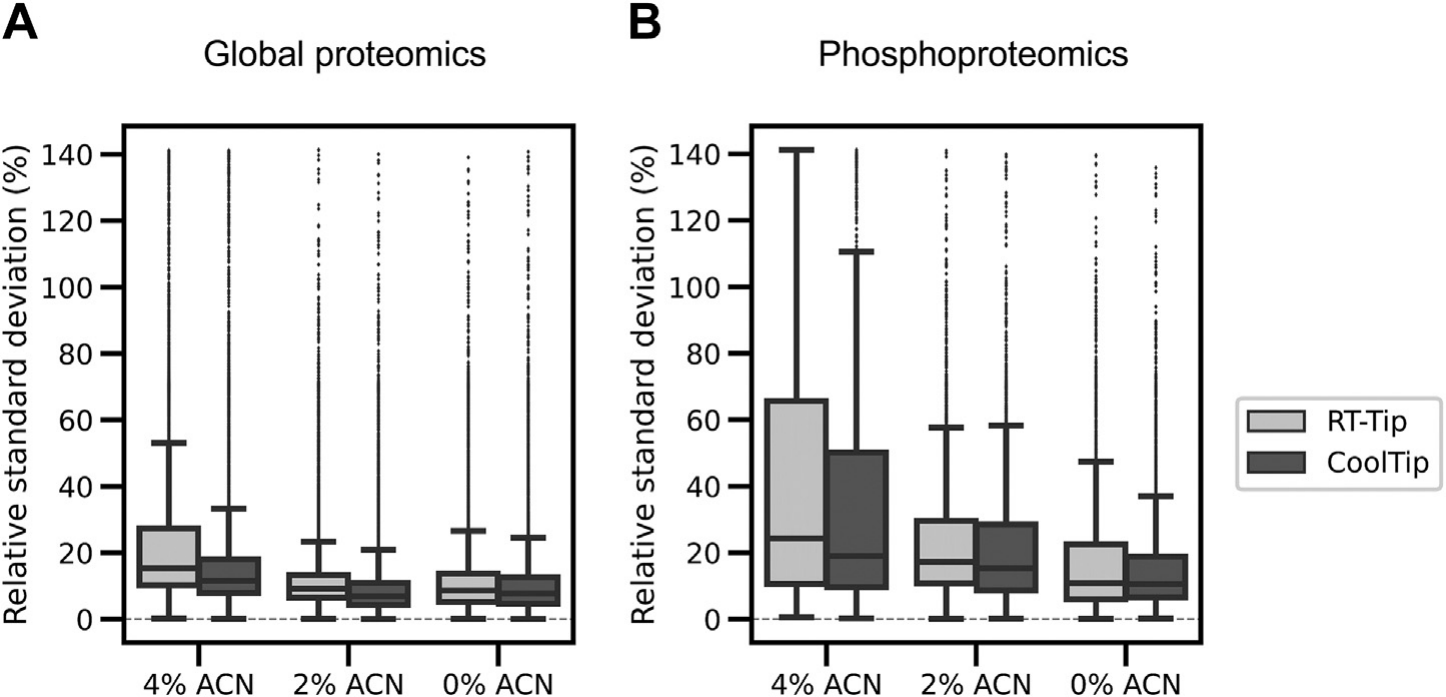
**Distributions of reproducibility of quantified peptides from triplicate analyses.** The *box* spans the interquartile range. The whiskers represent the 5% and 95% quartiles. The *thick horizontal line* in each *box* indicates the median. *A*, results from global proteomics. *B*, results from phosphoproteomics.

To quantitatively assess the recovery of peptides in the CoolTip protocol, label-free quantification was performed based on the MS1 precursor ion signals. The peak area information of identified peptides was extracted with the in-house match-between-run function, based on the targeted extraction of ion chromatogram (extracted ion chromatography) approach (36). We focused on peptides that were quantified across all six runs (triplicate analyses of RT-Tip and CoolTip) and calculated the peak area ratios of commonly identified peptides between the two protocols for each ACN concentration condition. As a result, better recovery was obtained with CoolTip (elution time <20 min), as shown for hydrophilic nonphosphopeptides in Figure 3, *A*–*C* and for phosphopeptides in Figure 3, *D*–*F*. All quantified peptides are summarized in supplemental data, located in the jPOST repository with PXD028871. On average, the recoveries of peptides and phosphopeptides at elution time <20 min were increased 2.1 times and 1.9 times in the CoolTip condition, respectively. Notably, the use of CoolTip did not result in a significant decrease of hydrophobic peptides, supporting the general applicability of the CoolTip protocol. Next, we evaluated the precision in triplicate analyses of global proteome and phosphoproteome samples using these two protocols. Relative standard deviation values of the obtained peak areas for identified peptides and phosphopeptides are shown in Figure 4, *A* and *B*, respectively. Better precision was obtained at lower ACN concentrations, which can be explained by stronger retention on RP materials in the presence of lower ACN concentration, leading to reduced loss of peptides during desalting. Similarly, the CoolTip protocol showed lower relative standard deviation values than the RT-Tip protocol, indicating that CoolTip also enables stronger retention, thus decreasing peptide loss during the desalting step. The CoolTip protocol does not require any special reagents and could be easily implemented in many kinds of shotgun proteomic experiments. Cooling the StageTip can be performed with a widely available refrigerated centrifuge, and it is not necessary to control the temperature of the LC/MS/MS system. Thus, the CoolTip protocol could be easily combined with the recently commercialized speLC/MS/MS system, which is designed to provide an interface between StageTips and a capillary LC system (37, 38) that increases the robustness of the analyses.

## Conclusion

We have developed a new desalting protocol that simply involves cooling the StageTip. We have shown that the CoolTip protocol extends the identification coverage of hydrophilic (phospho)peptides without sacrificing the recovery of hydrophobic peptides and also provides greater precision in quantitative analysis of the peptides. We believe this protocol would be easily applicable to improve the performance of many kinds of proteomics experiments.

## Data availability

The MS raw data and analysis files have been deposited at the ProteomeXchange Consortium (http://proteomecentral.proteomexchange.org) *via* the jPOST partner repository (https://jpostdb.org) with the dataset identifier PXD027112 and PXD028871. Supplemental data are also in the jPOST repository (PXD028871).

## Supplemental data

This article contains supplemental data.

## Conflict of interest

The authors declare no competing interests.
