# Supplementary material for: CoolTip: Low-Temperature Solid-Phase Extraction Microcolumn for Capturing Hydrophilic Peptides and Phosphopeptides
Source: Mol Cell Proteomics. 2021 Nov 3;20:100170. doi: 10.1016/j.mcpro.2021.100170 (PMC8646264; doi:10.1016/j.mcpro.2021.100170)
Supplement: Supplemental data [file mmc1.docx]

**Supplemental Data**

**CoolTip: Low-Temperature Solid-Phase Extraction Microcolumn for Capturing Hydrophilic Peptides and Phosphopeptides**

Kosuke Ogata^1^ and Yasushi Ishihama^1,2,*^

1) Department of Molecular & Cellular BioAnalysis, Graduate School of Pharmaceutical Sciences, Kyoto University, Kyoto 606–8501, Japan

2) Laboratory of Clinical and Analytical Chemistry, National Institute of Biomedical Innovation, Health and Nutrition, Ibaraki, Osaka, 567-0085, Japan.

*Corresponding author:

Tel: +81-75-753-4555, Fax: +81-75-753-4601, E-mail: yishiham@pharm.kyoto-u.ac.jp

--------------------------------------------------------------------------------------------------------------------

**Table of contents**

**Protocol for CoolTip desalting.**

**Figure S1.** Summary of total peptide identifications using different sorbents and ion-pair reagents.

**Figure S2.** Summary of peptide identifications with a series of StageTip temperatures.

**Figure S3.** Summary of peptide identifications using porous graphitic carbon tip (PGC-Tip) and CoolTip.

**Table S1.** Summary of the identified peptides. This file is located in the jPOST repository (<https://repository.jpostdb.org>) with PXD028871.

**Table S2.** Summary of the quantified peptides. This file is located in the jPOST repository (<https://repository.jpostdb.org>) with PXD028871.

**Protocol for CoolTip desalting**

**# Step 1 <Sample preparation>**

1. Perform protein digestion / phosphopeptide enrichment.

2. Reconstitute peptides in 0.1% TFA solution at a concentration less than 1 μg / μL.

**# Step 2 <StageTip preparation>**

**Materials:** SDB-XC: Empore™ SDB-XC Extraction Disks (CDS)

200 uL tip: 200 uL Diamond Tip (Gilson)

Syringe needle: Kel-F hub (KF), point style 3, gauge 16 (Hamilton)

1) Stamp out three pieces of SDB-XC disks were with a syringe needle

2) Pack the disks into a 200 μL tip.

Note: Capacity of SDB-XC cut by 16G needle: up to 10 μg peptides / membrane

**# Step 3 <CoolTip desalting>**

**Materials:** Solution A: 0.1% trifluoroacetic acid (TFA)

Solution B: 0.1% TFA, 80% acetonitrile

1) Cool the centrifuge by setting the temperature at 4ºC.

2) Place the StageTip into the centrifuge and wait for 5 minutes.

3) Load 50 μL of Solution B on top of SDB-XC membrane and centrifuge at 1500 × g for 3 min.

4) Load 50 μL of Solution A on top of SDB-XC membrane and centrifuge at 1500 × g for 3 min.

5) Load the sample solution on top of SDB-XC membrane and wait for 1 minute, then centrifuge at 1500 x g for 3 min.

6) Load 50 μL of Solution A on top of SDB-XC membrane and wait for 1 minute, then centrifuge at 1500 × g for 3 min..

7) Load 50 μL of Solution B on top of SDB-XC membrane and wait for 1 minute, then centrifuge at 1500 × g for 3 min.


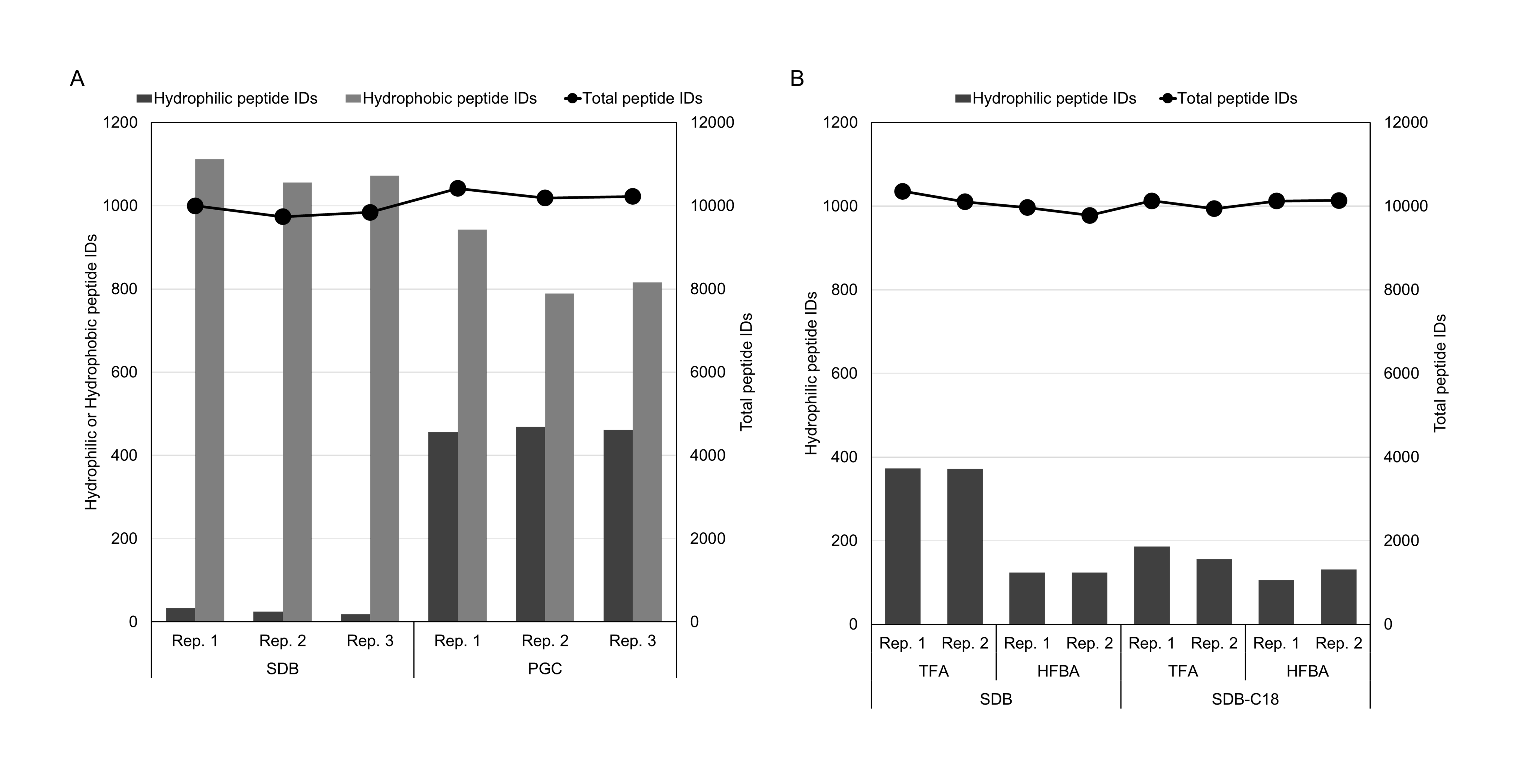


**Figure S1. Summary of total peptide identifications using different sorbents and ion-pair reagents.** (A) SDB-XC StageTips and PGC-StageTips, (B) SDB-XC StageTips packed with 1 mg of chromatographic sorbents (InertSep PLS-2 (SDB) and InertSep RP-C18 (SDB-C18)) were used for desalting 20 µg of HeLa tryptic digests dissolved in 4% ACN / 0.5% trifluoroacetic acid (TFA) or 0.5% heptafluorobutyric acid (HFBA). A 1 µg aliquot of desalted peptides was injected to the nanoLC/MS/MS. Hydrophilic peptides: peptides eluted at earlier retention times (< 20 min, corresponding to ACN concentrations below 8%). Hydrophobic peptides: peptides eluted at later retention times (> 70 min, corresponding to ACN concentrations above 28%). The results of (A) triplicate and (B) duplicate experiments are shown.


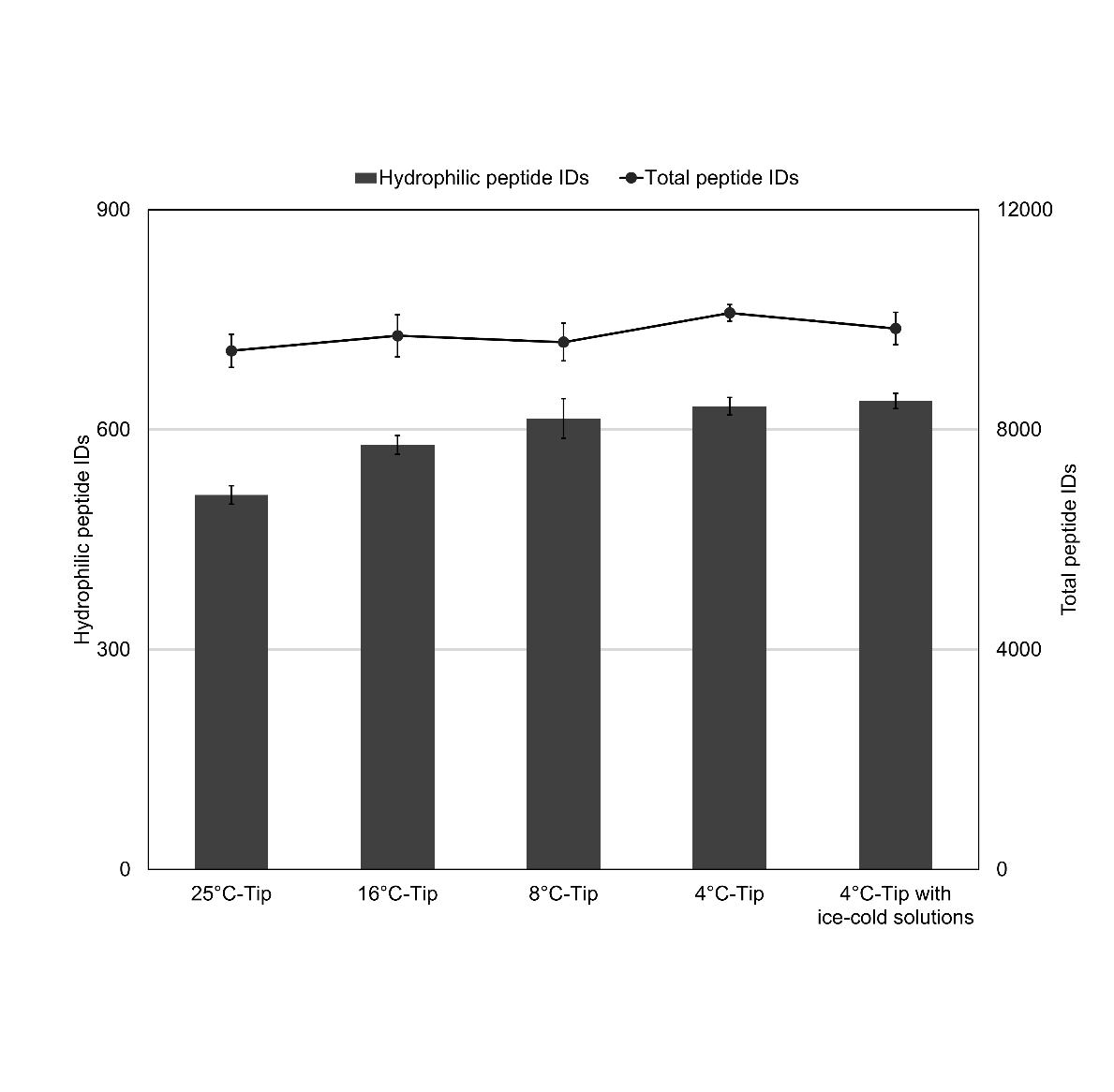
 **Figure S2. Summary of peptide identifications with a series of StageTip temperatures.** SDB-XC StageTips at 25 °C, 16 °C, 8 °C and 4 °C were evaluated with 5 µg of HeLa tryptic digests dissolved in 4% ACN / 0.1% trifluoroacetic acid (TFA). All solvents for sample loading, wash and elution were prepared at 25 °C or on ice. A 500 ng aliquot of desalted peptides was injected to the nanoLC/MS/MS. Hydrophilic peptides: peptides eluted at earlier retention times (< 20 min, corresponding to ACN concentrations below 8%). The average numbers from the triplicate experiments are shown. The error bars show the standard deviations.

**Figure S3. Summary of peptide identifications using porous graphitic carbon tip (PGC-Tip) and CoolTip.** An aliquot of 5 µg of peptides or around 150 ng of phosphopeptides was desalted using each StageTip. The 0% ACN solvent was used for loading and washing at the desalting step. Desalting using PGC-Tip was performed at room temperature. (A, B) Peptide identification numbers binned by retention time. The bar plots indicate peptide identification numbers in each bin. The error bars indicate the standard deviations of triplicate analyses. *: p < 0.05. (C, D) The bar plots show the average of total peptide identifications. The error bars show the standard deviations from triplicate analyses.
